# Involuntary Childlessness, Suffering, and Equality of Resources: An Argument for Expanding State-funded Fertility Treatment Provision

**DOI:** 10.1093/jmp/jhad026

**Published:** 2023-06-06

**Authors:** Giulia Cavaliere

**Affiliations:** King’s College, London, United Kingdom

**Keywords:** adoption, infertility, involuntary childlessness, resource allocation, same-sex couples

## Abstract

Assessing what counts as infertility has practical implications: access to (state-funded) fertility treatment is usually premised on meeting the criteria that constitute the chosen definition of infertility. In this paper, I argue that we should adopt the expression “involuntary childlessness” to discuss the normative dimensions of people’s inability to conceive. Once this conceptualization is adopted, it becomes clear that there exists a mismatch between those who experience involuntary childlessness and those that are currently able to access fertility treatment. My concern in this article is explaining why such a mismatch deserves attention and what reasons can be advanced to justify addressing it. My case rests on a three-part argument: that there are good reasons to address the suffering associated with involuntary childlessness; that people would decide to insure against it; and that involuntary childlessness is characterized by a prima facie exceptional kind of desire.

## I. INTRODUCTION

The World Health Organization estimates that about 10% of women in reproductive age (15–49) worldwide suffer from infertility.^1^ This estimate is based on data collected on women who have been in a stable relationship for 5 years and who have actively tried to conceive by performing regular unprotected intercourse, but have been unsuccessful. Reducing the timeframe to 2-years in a stable relationship, increases the prevalence of infertility in the female population by 2.5 times (WHO, 2021). Other studies relying on a similar timeframe estimate the prevalence of infertility in women to be between 6.6% and 26.4% (Boivin et al., 2007). In addition to the timeframe, other criteria to estimate the prevalence of infertility hinge on the (undefined) category of “regular” intercourse as well as the maintenance of the “propositional attitude” of trying to conceive, both of which are not clear-cut (Kukla, 2019). The criteria used to define infertility render this condition “identifiable and operationalizable” (Kukla, 2019) and influence data on its prevalence (Brown et al., 2016; Kukla, 2019).Whether everyone who is unable to conceive can therefore be considered “infertile” is a complex epistemic and normative question. It is a question that hinges on how infertility is defined. Moreover, it enables discrimination between forms of infertility that are addressed through state-funded fertility treatment and those that are not. Assessing what counts as infertility thus has tangible implications beyond estimating its prevalence: access to (state-funded) fertility treatment is premised on meeting the criteria that undergird the chosen definition of infertility (Brown et al., 2016; Kukla, 2019).

Among the countries that offer publicly-funded fertility treatment,^2^ some place statutory limitations to access such treatment. For instance, female age is one such limitation in many countries (Calhaz-Jorge et al., 2020).^3^ Another statutory limitation concerns the existence of previous children, which, for many countries, amounts to an exclusion criterion to access state-funded fertility treatment. In practice, many countries that offer state-funded fertility treatment place additional limitations to statutory limitations, such as those concerning female age, marital status, and sexual orientation, and curb the number of cycles that these, and other groups, can access (Calhaz-Jorge et al., 2020).^4^ This article canvasses whether these limitations—and the epistemic and normative reasons grounding them—are morally justified.^5^

I have structured my discussion as follows: in the first section, I explain why it is preferable to use the expression “involuntary childlessness” to refer to people’s inability to conceive unassisted. I then move to my *prima facie* case for addressing the mismatch between those who experience involuntary childlessness and those that are currently able to access fertility treatment. My case rests on a three-part argument: that involuntary childlessness causes a kind of suffering that we have good reasons to address; that involuntary childlessness is rather widespread and people might have good reasons to insure against it; and that involuntary childlessness is characterized by the desire for a certain kind of intimacy, building a certain kind of identity, and taking part in a certain web of social relations. In closing, I consider potential objections to my argument: the “adoption” objection and the “oppressive social norms” objection. Rather than being defeating objections, however, I explain that they help strengthen my case and should be factored in discussions on involuntary childlessness.

Let me to add a note before proceeding. Within this paper, I use the umbrella term “fertility treatment” to refer to the most commonly funded form of fertility treatment: IVF treatment. When discussing fertility treatment for older women, single women and women in same-sex relationships, I extend the scope of my analysis to include IVF treatment with oocyte (in the first and second case) and sperm (in the third case) donation. In all these cases, only one party would share a genetic link with the child. The argument I am advancing in this paper is that state-funded IVF treatment provision should be extended to include groups that are hitherto excluded from accessing such treatments.^6^

## II. INFERTILITY AND INVOLUNTARY CHILDLESSNESS

In addition to the difficulties associated with assessing the prevalence of infertility, estimating the number of women and couples who might *want to* access fertility treatment is similarly complex and likely to be dependent on a multiplicity of contextual issues, including economic and legal considerations. Boivin et al. (2007) calculate that between 42% and 73.3% of infertile couples in the Global North have sought to access fertility treatment. Percentages are slightly lower among infertile couples in the Global South, where there are more barriers to access (state-funded) fertility treatment, ranging between 27% and 74.1% (Boivin et al., 2007). Among those who seek to access fertility treatment, there are, for instance, women whose fallopian tubes have been removed; women who present with ovulatory defects; and men whose reproductive system shows signs of chromosomal or hormonal abnormalities. These people fall within a specific category of fertility treatment users: something is anatomically or biologically “wrong” with either them or their partners, and *this* explains their inability to conceive. But others who seek to access fertility treatment might do so for reasons that are not directly linked to anatomical or biological abnormalities of their reproductive system. For instance, couples whose infertility could be linked to immunological blood group incompatibility are unable to conceive *with one another*. Following the hypothesis of immunological blood group incompatibility, there is nothing inherently anatomically or biologically “wrong” with either party of the couple. They would counterfactually be able to conceive, were they trying to conceive with someone else. Another instance of perfectly anatomically and biologically normal people who seek to access fertility treatment are those whose infertility cannot be traced back to any recognizable cause. Their inability to conceive would be classified as “unexplained infertility” (Habbema et al., 2004), a euphemism for “we cannot find anything wrong with you, except that you seem to be unable to conceive”.

The idea here is that those who currently seek access to fertility treatment are a heterogeneous group whose infertility can be explained by a multiplicity of causes beyond anatomical or biological abnormalities. Additionally, there are also people who do not feature in estimates of fertility treatment users despite their inability to conceive unassisted, for they are excluded from accessing (state-funded) fertility treatment.^7^ For instance, anatomically and biologically “normal” people might not be able to conceive because of age-related infertility. Their infertility has biological causes (such as diminished ovarian reserve or sperm/oocyte quality), but their reproductive system functions “normally” for people in their age group. While women whose inability to conceive is linked to chromosomal abnormalities or hormonal imbalances are generally able to access (state-funded) fertility treatment, older women are often either legally excluded from accessing treatment or fail to meet the criteria to access it on publicly-funded healthcare systems. Further, single women, same-sex couples, overweight women, or smokers might be equally unable to conceive unassisted. As in the case of older women, these groups can be either legally excluded from accessing fertility treatment, or permitted to do so only privately, making access to treatment dependent on their financial circumstances.

Criteria to access fertility treatment are thus grounded on a definition of infertility that clearly demarks those who have a legitimate claim to access such treatment and those who fall outside its remit. An instance of a widely endorsed definition of infertility that uphold such demarcation is the World Health Organization’s definition, which states that infertility is “*a disease* of the reproductive system” (2021, emphasis added). Following this approach to defining infertility, people with certain anatomical or biological abnormalities of the reproductive system would be considered “infertile,” whereas same-sex couples, older and single women, would not be recognized as such. This approach thus draws a distinction between certain biological/anatomical causes underlying people’s inability to conceive and other causes to discriminate what legitimately counts as “infertility.” However, as Quill R Kukla notes, “the various attempts to define a coherent disease are an ontological, epistemological, and conceptual mess, with lacunae filled by ideological and social norms and meanings at each turn” (2019, 4417). Rebecca Brown et al. echo this in their contention that “the question of whether or not subfertility is considered a disease is a distraction” (2016, 291). To address this, they suggest focusing instead on “the harms resulting from subfertility, in particular the quality and extent of suffering it can cause, and how it affects individuals’ opportunities to pursue valued life projects” (Brown et al., 2016, 291). In a similar vein, the European Parliament’s recently approved *Report on the situation of sexual and reproductive health and rights in the EU, in the frame of women’s health* states:

Fails to encompass the reality of lesbian and bisexual women, as well as transgender persons, in same-sex couples, or single women interested in fertility options, *exacerbating the socio-legal challenges they already face in access to Assisted Reproductive Technologies (ART)* as a result of the focus on countering infertility. (Committee on Women’s Rights and Gender Equality, 2021, emphasis added)

At the same time, “lesbian and bisexual women may be unable to prove their ‘infertility’ and therefore be denied access to ART” (Committee on Women’s Rights and Gender Equality, 2021).^8^ My approach in this paper builds on these commitments, and considers what matters in recognizing a person as “infertile.”^9^ I will argue that the focus of discussions on the criteria to discriminate access to fertility treatment should move away from “infertility” and towards “involuntary childlessness”. This expression emphasizes that what matters with respect to people’s inability to conceive is the lack of the “end product” (they are *childless*)^10^ and the dispositional attitudes surrounding such a state (they are childless *involuntarily*). Following this approach, the woman whose fallopian tubes have been removed and the woman who is single but would like to have children are both to be considered involuntarily childless, for they both cannot achieve genetic parenthood unassisted. Employing “involuntary childlessness” as a category to identify the groups that would access (state-funded) fertility treatment in a counterfactual world where they were permitted to do so, reveals that there exists a mismatch between the number of people who experience involuntary childlessness and the number of people allowed or able to access (state-funded) fertility treatment.^11^ Establishing why this mismatch is problematic and examining the justifications that can be advanced to expand criteria to access fertility treatment (and thus address the mismatch), is my concern in this paper.

## III. DISCRIMINATING ACCESS TO FERTILITY TREATMENT

As mentioned in the previous section, for the purpose of this paper, I use the expression “involuntary childlessness” to characterize people’s inability to conceive unassisted (if they wish to do so). A note before proceeding: my aim in this paper is to argue for addressing the mismatch between people who can currently access fertility treatment and people who similarly suffer from involuntary childlessness but are hitherto excluded from accessing it. It is not to settle philosophical discussions on whether infertility counts (or should count) as a disease.

Brown et al. (2016) note that *some* (anatomically and biologically) infertile people prefer definitions of infertility that conceptualize it as a disease, for the disease-status of infertility is thought to aid demands to allocate medical resources to treat it. That is, allocation of medical resources to the treatment of a specific condition is often premised on the recognition of such a condition as a *disease*. The treatment for Parkinson’s has, thus, more chances to be allocated (medical) resources than that for loneliness, even if both are likely to negatively affect people’s well-being and their lives. While the disease-status of many conditions is a matter of debate, bio-statistical accounts of health and disease ground demands for (medical) resource allocation.^12^ Despite this, in the case of infertility,^13^ I consider such conceptualization problematic for two reasons. First, as discussed in the previous section, current definitions of infertility such as the WHO definition pay lip service to the bio-statistical model by defining this condition as a disease. At the same time, there are epistemic and normative considerations at play in defining infertility and in operationalizing it as a boundary-making concept, as noted by Kukla (2019). Second, as I argue in this section, the reasons that ground offering fertility treatment to a woman whose fallopian tubes have been removed (and who wishes to have children, but is unable to do so unassisted), and not to a woman who is single, older, or in a same-sex relationship (and who wishes to have children, but is unable to do so unassisted) are contentious, for they rest on what I consider to be an arbitrary distinction between cases that does not track what fertility treatment addresses.^14^

Allow me to explain. What fertility treatment addresses in the case of the woman whose fallopian tubes have been removed, is not her anatomical abnormality, but her inability to conceive (if she wishes to do so). Her anatomical abnormality would not be medically addressed in a counterfactual world where she did not wish to have children. Rather, fertility treatment addresses her inability to have children in a world where she wants to have children. It gives her a chance to fulfill her parenthood project. Now, this is true both in the case of the woman whose fallopian tubes have been removed *as well as* in the case of the single woman (and the older woman or the woman in a same-sex relationship). For what the fertility treatment does in this latter case, is not to address her singlehood, age, or sexual orientation, but the implication for her parental project of her relationship status and age.

At this point, one could argue that resources are limited and discrimination between different cases (and causes) is necessary. But, for discrimination to be justified, the reasons to discriminate should be legitimate. What, then, justifies favoring someone whose fallopian tubes have been removed and is thus unable to conceive over someone with both fallopian tubes in place, but who does not have a partner and is thus unable to conceive? Both women are unable to conceive unassisted and both women might be negatively affected as a result, hence their seeking access to fertility treatment. Grounding access to fertility treatment on the disease-status of infertility is based on a distinction between the reasons to provide fertility treatment that is question begging, in that it relies on the very thing that it is supposed to prove: that infertility is a disease of the reproductive system that needs to be treated. As Kukla puts it: “infertility thus purportedly deserves treatment because it is a disease, and it is a disease because it is recognized as one” (2019, 4422). Moreover, it fails to track the reason infertility is problematic, weakening instead of strengthening the legitimacy of demands to allocate medical resources to treat infertility. It is for these reasons that I contend that the focus should be on involuntary childlessness rather than on infertility: it is inclusive, and it adequately tracks what makes infertility an undesirable state.

## IV. INFERTILITY AND SEVERE AND ENDURING SUFFERING

The first part of my argument in favor of addressing the mismatch between the people who can access (state-funded) fertility treatment and people who are hitherto unable to do so, is that both groups suffer as a result of not being able to conceive unassisted. My argument relies on two premises. First, I contend, but do not argue, that other things being equal, reducing people’s (severe and enduring) suffering is a morally desirable thing to do. Second, my argument is conditional on an empirical question concerning people’s suffering. That is, if people’s suffering from the experience of involuntary childlessness is severe and enduring, and fertility treatment can contribute to reducing that suffering, then we have *pro tanto* reasons to expand access to such treatment,^15^ given the first premise.^16^

While collecting data on the psychological effects of infertility presents methodological challenges,^17^ qualitative studies on this matter show that there is a correlation between the experience of infertility and psychological distress and suffering. Women are generally reported to be more affected than men or affected in different ways,^18^ mediated by broader social norms (Throsby and Gill, 2004). Infertility becomes a negative identity (Bell, 2019) and assumes a central role in people’s lives (Johansson and Berg, 2005). It has adverse effects on the way they experience personal and social relationships and contributes to feelings of isolation (Parry and Shinew, 2004). Reported feelings from people who are unable to conceive relate to extreme sadness, frustration, anxiety, stress, and a sense of worthlessness and inadequacy (McQuillan et al., 2003). Women also experience self-blame, even when the cause of infertility is unknown (McLeod and Ponesse, 2008). What emerges from these accounts is that people’s sense of self (their identity) and well-being are compromised by the experience of infertility, which, for many, constrains the identity-building project of parenting. While such feelings are neither universally experienced by childless people, nor by involuntary childless people,^19^ evidence from empirical studies of infertility show that the suffering that people experience is widespread, severe, and enduring. This is, in my view, the first *prima facie* argument to address the mismatch between the people who would like to access fertility treatment and the people who actually access fertility treatment, for their suffering and the reasons underlying it are the same.

Now one could argue that fertility treatment’s efficacy varies, and accessing treatment often does not translate into the achievement of a viable pregnancy/birth. This is true both in the case of people who experience anatomical and biological forms of infertility, as well as other forms of infertility, such as those described at the outset of this paper. Moreover, success rates of fertility treatment often correlate with the number of cycles of such treatment offered to women and couples. One way to address this and to increase the chances of success would be to enable the delivery of a number of cycles that is considered clinically (and cost-)effective. Alternatively, one could object that offering medical assistance for what can be conceived as a social problem further medicalizes an existential state. Again, this is true in the case of people whose infertility is anatomical or biological, for treatment does not address their anatomical or biological abnormalities, but their desire to have genetically related children. I am of the view that saying that something becomes increasingly medicalized does not *per se* track its problematic nature. It is what medicalization can cause, that is, adverse effects on people’s life and well-being, that can be problematic. I return to this issue below, where I consider objections to my argument. Relatedly, using suffering as a justification to increase access to fertility treatment is often countered by the idea that women’s (and, to a lesser extent, men’s) yearning and suffering can be caused or at least worsened by social norms concerning women’s role in society; the primacy of the nuclear family over other forms of kin relations; the emphasis of genetic relatedness; and pronatalism. These norms are said to harm women—both women who experience involuntary childlessness and those who do not—because they shape their desires and behaviors in oppressive ways. I extensively consider such an objection below. For now, it is important to note that even if such suffering is socially constructed, *this* does not make it any less real to the people who are experiencing it. Nor, given the entrenched and established nature of social norms concerning women’s role in society and parenthood, is it easy to do away with.

## V. INFERTILITY AND THE INSURANCE MARKET IDEAL

In the previous section, I have argued that there are good reasons to believe that the frustration of people’s parental project is linked to widespread, severe, and enduring suffering. This, however, is only the first part of my argument for addressing the mismatch between those who are able to access fertility treatment and those who are hitherto unable to do so. That is, it amounts to a necessary but not sufficient condition to address such a mismatch. The frustration of other projects might produce a similarly severe and enduring suffering. What, then, makes this particular project worthy of investing resources? In the present and the following sections of this paper, I am concerned with showing that the frustration of this particular project warrants the investment of resources necessary to address the mismatch. To do so, I build on Ronald Dworkin’s insurance market for healthcare rationing^20^ and on specific aspects of the preference for a certain parental project.

Estimating the actual size of those who would seek access to (state-funded) fertility treatment if they were able to do so is complex. Many of the people who experience involuntary childlessness might not seek treatment due to existing statutory and financial barriers and might thus be overlooked in national and international estimates of those seeking to access such treatment. For instance, while there is empirical and anecdotal evidence of single women or same-sex couples who wish to have children and would rely on fertility treatment to do so, they might not seek treatment, due to such barriers. These estimates are thus *conservative* if one conceives infertility as involuntary childlessness. As discussed above, it has been estimated that infertility affects between 6% and 26% of the female population worldwide, depending on the adopted definition of infertility and on the study sample and methods. These estimates show that people’s risk of infertility is substantial, even granted the conservative nature of such estimates. As with the first part of my argument, the second part similarly relies on two premises, the first being that, as Dworkin puts it, I take the “demands of equality” to be “prior to other desiderata” (1981, 295). That is, I consider treating people equally as an important good, one that we have good reasons to promote. Second, my argument in this section is again conditional on the truth-status of my hypothesis concerning involuntary childlessness. My hypothesis is that, considering the prevalence and incidence of involuntary childlessness and the (severe and enduring) suffering associated with it, there are good reasons to believe that people would opt for including fertility treatment within the set of treatments provided by a publicly-funded healthcare system.

Dworkin’s discussion of healthcare rationing builds on his broader theory of equality as equality of resources.^21^ Medical resources are in this sense one among many sets of resources that, if allocated justly, contribute to people’s equality. Dworkin (1981), in his “prudent insurance” ideal applied to healthcare rationing, asks us to imagine a society where resources are distributed fairly; where people are aware of their preferences and tastes; where they have knowledge about the costs of medical procedures; the likelihood of diseases to occur and their severity; and where people do not have any knowledge of their own specific risks of contracting any particular disease. According to the approach that Dworkin proposes, fair distribution of (medical) resources mirrors the hypothetical decisions that rational agents make in ideal conditions.^22^ Rational agents who possess the kind of information just outlined would come together to deliberate on two questions: how much of the available funds that they have to spend on social needs should be devoted to health-related needs, and what services should basic healthcare coverage include. Now what is Dworkin’s hypothesis as to what would be included in that package? Under these conditions, each person would decide what is prudent for them to have in such a package, considering the likelihood of a certain condition to occur, and its severity, but also their personal preferences and inclinations. The aggregate results of this deliberation would then serve as a blueprint for what a basic insurance package would cover. Some people might have special preferences: they might want to insure against color-blindness or, borrowing from Dworkin, costly medical care at the end of their lives at the expense of medical services that could be offered throughout their conscious lifespan. In other words, some people might have what can be considered “eccentric tastes” (Dworkin, 1981). What to make of them? Dworkin argues that the insurance market ideal provides a simple answer to this question. That is, a package that includes the eccentric tastes of the few is unlikely to be purchased by the many, for it would fail to match their preferences, needs, and values. As a result, it would not be a good candidate for the insurance market, which self-regulates against such tastes.

Ex hypothesis, involuntary childlessness is something that would be included in a package purchased by many, if rational agents were to deliberate on this issue. This is due to the sheer number of people that experience it (or, following Dworkin’s approach, would be at risk of experiencing it); the suffering associated with involuntary childlessness; and the value that people seem to place on the fulfillment of parental projects.^23^ But, if this is true for those who can currently access fertility treatment, I argue that it is equally true for those who are either legally excluded from accessing treatment or whose access to treatment depends on their purchasing capacity. This is because of the characteristics of the latter group’s parental project vis-à-vis the former group’s project: both groups are similarly experiencing a shortfall in their personal resources. It would therefore be fair to include fertility treatment within the services that the hypothetical insurance market would cover. This is the second part of my argument for addressing the mismatch between those who may wish to access fertility treatment and those that are hitherto able to do so.

At this point, one could counter my conclusion by arguing that older women, same-sex couples, and people whose lifestyles can partially explain their infertility (such as smokers or overweight women) can be considered to be responsible for their involuntary childlessness in a way that women with chromosomal abnormalities cannot.^24^ The latter kind of involuntary childlessness, unlike the former, is a product of brute luck.^25^ Whether people who have postponed the time of (attempting) conception or people whose inability to conceive can be partially^26^ tracked back to their lifestyles are responsible for such lifestyles is a complex question, one that hinges on discussions within luck egalitarianism about personal responsibility and luck,^27^ as well as on the scholarship on the social determinants of health and disease. The core idea here is that there are certain kinds of disadvantages (such as involuntary childlessness due to age-related infertility or lifestyle) for which people should not be held responsible, because the causal mechanisms that contribute to their emergence cannot be solely tracked back to choices that people have deliberately made.^28^ Rather, such mechanisms involve individual decisions and behaviors *as well as* socio-economic and political circumstances that constrain the decisions and behaviors of individual agents. Material and immaterial circumstances have great influence on people’s health outcomes, including their reproductive potential. And this is true in the case of involuntary childlessness due to lifestyle as well as in the case of involuntary childlessness due to age-related infertility. Lifestyle-related diseases, and the poor health outcomes with which they are associated, are more common among disadvantaged groups; the mechanisms that influence their emergence, are often influenced by social and cultural environments, occupational status, wealth, education, and other factors. Similarly, as I have argued elsewhere with respect to age-related restrictions to access fertility treatment, “the global phenomena of reproductive aging, whereby the timing of the first child moves further along the life course, is heavily politically, economically, and socioculturally constrained” (Cavaliere and Fletcher, 2022, 1000). My contention is that women are often deemed personally and morally responsible for age-related infertility, and that this overlooks both these women’s circumstances and broader socio-political trends, such as, for instance workforce feminization that structurally determined these women’s ability to conceive unassisted.

## VI. INFERTILITY AND PEOPLE’S DESIRE FOR A CERTAIN PARENTAL PROJECT

In the previous sections, I have argued that we have *pro tanto* reasons to relieve people’s severe and enduring suffering; and that, considering the prevalence of involuntary childlessness, and the suffering associated with it, it is at least plausible to foresee a scenario wherein rational agents would allocate (medical) resources to address such a condition. These have been the first two parts of my argument for addressing the mismatch between the people who can access fertility treatment and those who are hitherto unable to do so. The third part of my argument rests on certain characteristics of the desire for the parental project of having (genetically related) children. In this last section, I am hence concerned with showing that the desire for a certain parental project should be considered *prima facie* exceptional with respect to other desires, due to the characteristics of such a desire. These characteristics distinguish the desire for a certain parental project from other desires that people might have and other life-projects that they might want to pursue. I will argue that the desire to have one’s own children is a desire for the kind of intimacy that is not immediately replicable in other meaningful relationships; for changes in people’s identity; and for their participation in certain webs of relations.

Building on Ferdinand Schoeman’s (1980) account of parental rights, Harry Brighouse and Adam Swift argue that there are significant differences in the quality of the intimate relationships that adults can develop with one another and with the children that they parent.^29^ According to them:

The relationships that adults have with the children they parent are not merely additional intimate valuable relationships, which contribute to their flourishing in the same way as their relationships with other adults. They have a different moral quality, make a different kind of contribution to their flourishing, and so are not interchangeable with other relationships (Brighouse and Swift, 2006, 92).

The idea here is that intimacy and building intimate relationships contribute to people’s flourishing. Moreover, the intimate relationship with one’s children is qualitatively different from other relationships. Unlike the love that one can receive in relationships with other consenting adults, children’s love for their parents, at least in the first few years, is ‘“spontaneous and unconditional,” and “outside the rational control of the child” (Brighouse and Swift, 2006, 93). The intimacy developed in early years is also about co-dependence, where the parents (or parent) are the only means for the child’s survival. Again, this is not typical of most other intimate relationships.^30^

In addition to this, the parental project is also a desire for the coming into being of a new identity. Fertility treatment, as Charis Thompson (2005) puts it, “makes parents,” not just babies. Identity-building arguments, especially when they relate to motherhood, can be used in oppressive ways. That is, the association between (those who identify as) women and the social role of “mothers” has been used to constrain women’s choice into one single valuable project: becoming mothers. Social norms concerning fatherhood and motherhood, as well as biological changes in women’s and men’s bodies, shape people’s identity when they transition to these new social roles.^31^ This can occur in oppressive ways.^32^ For example, when women are primary carers and, as a result, forego other valuable life-projects. Or, when “father” becomes synonymous with “breadwinner,” constraining men’s agency and mediates, often negatively, their interaction with their children. But the desire to have children is also the desire to embody and experience these identities in non-oppressive ways, to *become mothers and fathers*. This is ultimately the promise of fertility treatment: to enable the formation of a new identity, which, at least in the abstract, turns around the negative identity often associated with the lack of the child wanted and desired, the experience of involuntary childlessness.

Lastly, whilst involuntary childlessness is often characterized by loneliness and feelings of exclusion, having children enables individuals to enter new webs of relations. It enables people to socialize with other parents; to take part in rituals and ways of living associated with having and rearing children; and to be socially recognized as part of a community, that of parents and grandparents. This happens at the micro level of conversations, shared stories, school runs, baby showers, and other instances of socializations with other parents, as well as at the macro level, where social policy is often framed around family-units and heteronormative, mononuclear families.

To the critical, feminist, and politely skeptical reader, many of these reasons to address the mismatch between those who can access fertility treatment and those who are hitherto unable to do so might reek of heteronormativity, reactionary attitudes, and sexist social norms. That is, proposing to expand access to fertility treatment as a “solution” to the suffering; negative identity, and desire to fulfill a certain parental project that entails having genetically related children might suggest that the problem lies in people’s inability to procreate, rather than in pronatalist, heteronormative, and sexist social norms that reinforce the importance of genetic ties, of having children, and of women’s role in procreation. This is not my intention, and this is a set of critiques that I take seriously and discuss in the next section, together with other objections that can be raised against the three-part argument defended in this paper.

## VII. OBJECTIONS

A number of objections can be raised against my argument in favor of expanding access to fertility treatment. Many of these objections, however, succeed in debunking one part of the argument, whilst falling short on succeeding against the whole three parts taken together.^33^ Here, I restrict my discussion to two objections that engage with broader problems with expanding access to state-funded fertility treatment: the adoption objection and the oppressive norms objection.

The first objection, which has been raised often in the philosophical literature on assisted conception, considers that fertility treatment is not the only means to fulfill a parenthood project: people could adopt instead (Rulli, 2016). The idea is that fertility treatment is burdensome for the physical and psychological health of the woman who undergoes it (and psychologically burdensome for her partner too); expensive for the state, and has rather low success rates. Moreover, and importantly, there are many children in need of adoption. What, then, justifies creating children to fulfill people’s parental project? Tina Rulli (2016), for instance, examines the reasons to justify a preference for a genetically related child and observes that they fail to defeat a *pro tanto* duty to adopt rather than to create a child. “Why do they not just adopt?” is a compelling objection for the three reasons listed above: fertility treatment is emotionally and physically burdensome; it can fail, which often generates additional psychological stress and suffering; and there are indeed many existing children that need parents. The problem with this objection, however, is that it fails to take seriously people’s desires, the severe and enduring nature of their suffering, and the barriers associated with adopting children. It is an empirical question as to whether people’s (severe and enduring) suffering associated with involuntary childlessness could be alleviated by adopting existing children rather than trying to have genetically related children with fertility treatment. Aside from this, however, social workers and psychologists involved in the adoption process are adamant that adoption is *not* a replacement for the child an individual or a couple did not have due to failed natural or assisted attempts to conceive. That is, whilst adoption “makes parents,” it does so in a different way from fertility treatment. The relationship with the adopted child is not (and should not be) a replacement for the relationship with the genetically related child that one has failed to have. Moreover, people’s suffering associated with involuntary childlessness is not necessarily a kind of suffering engendered by the frustration of the desire to parent *any* child. Rather, it is a severe and enduring suffering associated with the impossibility to have one’s own, genetically related child.

The skeptic here is thus confronted with a dilemma: either one disregards people’s preferences and the suffering associated with the frustration of the desire for a genetically related child, or one honors such preferences; fails to provide a solution for children in need of adoption; and devolves resources that could be directed elsewhere to expanding fertility treatment provision. Both options, in other words, involve significant trade-offs. The problem with the first option, however, is that it places responsibility for alleviating the issue of children in need of adoption on involuntary childless people, rather than on everyone who wishes to fulfill their parenthood project. Moreover, it might not address people’s suffering either, for the “adoption alternative” carries over some of the existing barriers with accessing treatment. As discussed in the Introduction, some groups’ ability to access fertility treatment is conditional on statutory rules and regulations that either exclude them from being able to access such treatment or make their access dependent on their financial ability to do so privately. Hence, it is likely that these groups would have to shoulder the bulk of the responsibility engendered by a putative duty to adopt. Not only, as the Matić Report states, does this exacerbate existing challenges that they face in fulfilling their parenthood projects, but also *the nature of the adoption process* is not conceived in a way that accommodates difference. Single women, same-sex couples, people with low socio-economic status, and people of color are usually part of the group that is less likely to be eligible to access state-funded fertility treatment, but they often are part of the very same group that is unlikely to pass the close scrutiny and screening that comes with applications to adopt or foster children. Hence, the adoption objection rests on an unequal distribution of responsibilities and unequal concern for people’s preferences and suffering. Moreover, it presupposes rather than justifies, that changing the adoption process is more desirable than changing fertility treatment provision.

A further objection that can be raised against my argument concerns the role of social norms in shaping people’s desire to access fertility treatment and, crucially, the suffering associated with involuntary childlessness. The idea here is that the suffering associated with the frustration of people’s parental project, the desire to build a kind of intimate relationship such as the parent–child relationship, and the wish to embody the parental identity (and status), are by-products of pronatalist, social norms and of the pressure on women to fulfill their social role of mother (McLeod and Ponesse, 2008; Petropanagos, 2017). Relatedly, considering that involuntary childlessness can be conceived as an existential state shaped by social norms, it might be puzzling to consider fertility treatment, that is, *medical* treatment, as the solution for the suffering associated with such a state. Expanding access to fertility treatment does nothing to alleviate the pressure on women to procreate and to reduce the primacy of certain parental projects (such as having genetically related children) over others (such as adoption). Expanding access to such treatment to larger segments of society might have a performative effect.^34^ It might promote rather than challenge oppressive social norms and the suffering associated with the frustration of the preferred parental project.^35^ Moreover, fertility treatment is neither morally nor gender neutral. It can be physically and emotionally burdensome, and women shoulder the majority of such burdens. It entails invasive procedures that are performed largely on women’s bodies, such as hormonal stimulation, egg retrieval, and embryo transfer. The high rates of failed embryo creation and transfer and the frequent miscarriages that occur in such processes can potentially increase, rather than relieve fertility treatment users’ suffering.

One could argue, then, that considering the potentially negative effects on users and on society at large of expanding access to fertility treatment, efforts should be channeled elsewhere. For instance, to address the suffering associated with involuntary childlessness, the negative connotation of the childless identity, and the exclusion from social relations that childlessness often entails, efforts should be channeled into changing the social norms that influence the emergence of the demand for such treatment in the first place, rather than in expanding access to such treatment. As Sally Haslanger puts it:

Even granting the cultural significance of the natural nuclear family schema, there are two ways to combat this stigma. One is to provide resources so that everyone can come as close as possible to fitting the schema, another is to combat the dominance of the schema. Velleman prefers the former strategy; I prefer the latter. (2009, 119)

I agree with Haslanger: genetic parenthood—which is what fertility treatment gives involuntary childless people a chance to achieve—should not be considered “universal, necessary, and good.” But, for involuntary childless people, it is often experienced in that way. Combating the dominance of the schema by leaving unchanged or reducing (state-funded) access to fertility treatment is paternalistic, in that it fails to take involuntary childless people’s interests and preferences at face value. Rather, it is a strategy that is modeled on their purported objective interests.

Expanding access to fertility treatment to people who are hitherto not able to access it, however, need not be (and should not be) the end of the conversation on the ways to address involuntary childless people’s suffering. Medical solutions need not be the only solutions worth pursuing. The performative effects of this strategy and the potential long-term drawbacks of reinforcing certain social norms instead of questioning them should not be underestimated. It is for this reason that efforts to expand access to (state-funded) fertility treatment should go hand in hand with a reflection on how to resist oppressive social norms and work collectively to both reduce their impact on (mostly) women, as well as producing new, more liberatory norms. The choice does not need to be either fitting the schema or combating its dominance. Rather, what should be pursued is a combination of these two strategies: one that takes involuntary childless people’s suffering and preferences seriously by enabling their access to fertility treatment, whilst, at the same time, critically engaging with oppressive social norms and with the material conditions that sustain them.

## VIII. CONCLUSION

In this paper, I have shown that criteria to access state-funded fertility treatment rests on epistemic and normative considerations operationalized to draw boundaries between types of infertility that are addressed through state-funded fertility treatment, and types of infertility that are excluded from such treatment. I have argued that such considerations warrant further discussion and scrutiny, and that they rest on problematic assumptions. For this reason, I have canvassed the possibility that the suffering associated with involuntary childlessness; the prevalence of such a condition; the importance of equality; and, finally, certain characteristics of the desire to have genetically related children, warrant a rethink of state-funded fertility treatment provision. I have also considered some objections to my argument, which I see as complementary to the broadly egalitarian project in which I am invested rather than in opposition to it.

## Footnotes

1 Data on the prevalence of male infertility is more challenging to collect.

2 State funding in the Global South remains extremely limited (Morshed-Behbahani et al., 2020).

3 Upper limits to (female-)age vary significantly. For instance, countries such as Austria, Germany, and Sweden have such limit at thirty-nine whilst others such as the Czech Republic are at forty-nine (Calhaz-Jorge et al., 2020)

4 The number of publicly-funded cycles varies significantly across countries (Calhaz-Jorge et al., 2020), with the most common being a three-cycle limit. Countries such as Slovenia and Belgium have among the most generous funding with respect to the number of cycles, offering up to six cycles (Calhaz-Jorge et al., 2020). On the more conservative side of the spectrum, national guidelines in the UK recommend offering up to three cycles to women under the age of forty. Despite this, due to funding restrictions, IVF provision in the UK differs markedly from national guidelines (HFEA, 2021). Some clinical commissioning groups limit access to three cycles to women under thirty-six, whilst others offer only one cycle of treatment (HFEA, 2021). For a discussion of IVF funding and age-related infertility in the UK, see (Cavaliere and Fletcher, 2022).

5 My conclusions and arguments are not conceived with a specific healthcare system or jurisdiction in mind. They are, however, especially relevant for countries that already have state-funded IVF provision.

6 I do not discuss the possibility of expanding fertility treatment provision to single men and gay men in same-sex relationships. The reason for this is that for these groups to be able to have (partially) genetically related children, there is a need for more than just genetic material (in the form of oocytes) to be supplied by women. There is, trivially, the need for a woman who acts as a surrogate. Whilst it is true that oocytes donation is not without problems, if only that it places the burden of supplying genetic material on women, the need for gestational surrogates complicates the picture in a way that, in my view, would warrant a separate discussion. I am indebted to Teresa Baron and to an anonymous reviewer for pressing me to clarify this issue.

7 A note on this: in this paper, I use the expression “access to (state-funded) fertility treatment” as a short-hand for two scenarios: jurisdictions that exclude certain groups from accessing state-funded fertility treatment, and jurisdictions that exclude certain groups from accessing fertility treatment *simpliciter*.

8 I am grateful to one of the anonymous reviewers for bringing this Report to my attention.

9 As Arthur Greil et al. put it: “the presence of infertility is signaled, not by the presence of pathological symptoms, but by the absence of a desired state” (2010, 141).

10 I am aware that some of the people who seek to access fertility treatments have already had children. In this paper, however, I am concerned with people who are, in the moment they seek to access fertility treatment, childless.

11 This includes both people who are legally excluded from accessing fertility treatment as well as people who need to do so privately because they do not meet criteria to access state-funded treatment.

12 Such as Norman Daniels’ argument for justice in health care (Daniels, 2007).

13 As in other cases, as the scholarship that critically engages with bio-statistical definitions of health and diseases has widely shown.

14 An alternative strategy to support this idea is to argue that social factors should be given a similar weight to medical factors in decisions concerning the allocation of medical resources. As Stephen Wilkinson and Nicola Williams note, “there may be instances in which it is appropriate for the state to use its resources to address issues other than disease” (2016, 562). In a commentary to the Wilkinson and Williams article, Mianna Lotz similarly contends that: “decisions regarding public health funding ought not to treat social factors as automatic grounds for disqualification. So many of the harms of accepted ‘diseases’ and ‘disabilities’ turn out on closer analysis to arise in virtue of social factors, including desires, preferences, and priorities, rather than purely medical ones. This is part of what it means to say that notions of health and well-being, and their counterparts illness, disability, and disease, are inherently normative and evaluative” (2016, 570). My discussion in this section is both orthogonal and compatible with that of these authors.

15 The reasons are *pro tanto,* for there could be countervailing reasons not to expand access to such treatment. For instance, one could argue that not all forms of suffering justify (medical) intervention. In this sense, I consider that the suffering would have to be at least “severe” (qualitative condition) and enduring (chronological condition). Alternatively, one could argue that there might be other, more morally weighty issues that require attention and state-funded resources. Carrying out an evaluation of the (moral) priority that each issue might have over the suffering associated with involuntary childlessness is beyond the scope of the article, but I would accept this objection. The (severe and enduring) suffering associated with involuntary childlessness does not necessarily trump other forms of suffering in acquiring priority over state-funded interventions.

16 My contention is similar to Brown et al.’s. They argue that: “the particular forms of suffering and disruption to valued life projects associated with subfertility may justify some degree of state support, although the form such support takes need not be medicalized” (Brown et al., 2016, 295).

17
Pasch and Christensen (2000), among others (Greil, 1997; Greil et al., 2010), list some of the shortcomings in social-scientific research on infertility: small sample sizes, poor sampling methods, use of non-standardized measures, lack of adequate control groups, and studies being conducted in infertility treatment centers with which the researcher is affiliated.

18 For a review of studies on this, see Greil (1997)

19 It must be noted that the bulk of the studies cited in this article use samples drawn from fertility patient populations rather than from people who experience infertility who have not sought access to fertility treatment. As Greil et al. put it, “without a non-clinic comparison group it is impossible to untangle the effects of infertility from the effects of infertility treatment on psychological outcomes” (Greil et al., 2010, 143).

20 For a detailed account of how Dworkin’s theory of equality of resources can be extended to justify the public provision of assisted reproductive technologies, see Burley (1998).

21 For an extensive account of this theory, see Dworkin (1981).

22 That is, in a society where there is fair equality in the distribution of other (i.e., non-medical) resources, and where people have state-of-the-art knowledge comparable to that typically had by doctors.

23 For a comprehensive discussion of this claim within a Dworkinian framework, see Justine C. Burley’s (1998) paper, “The price of eggs: Who should bear the cost of fertility treatments?”

24 I am grateful to one of the anonymous reviewers for pushing me to clarify my position on this matter.

25 Interestingly, fallopian tubes’ inflammation, scarring, and blockage that cause infertility can also occur as a result of contracting sexually transmitted diseases (STDs), such as chlamydia and gonorrhea, especially when these conditions are left untreated. Arguably, such kind of infertility differs from the kind of infertility that is (straightforwardly) a product of brute luck, such as fallopian tube agenesis, which can be congenital. However, my argument in this section is that it is problematic in many instances to hold people morally responsible for STDs-related and other kinds of infertility. I am indebted to one of the anonymous reviewers for this example.

26 Partially because of the limited evidence that links certain lifestyles with infertility; see, for instance, Brown (2019).

27 On this issue, see especially Cohen (1989).

28 This also applies to sexual orientation, which I do not discuss here, because I believe it to be relatively uncontroversial that people’s sexual preferences are not to be considered the product of choices that people have deliberately made and for which they should be thus morally responsible.

29 Brighouse and Swift’s argument is about the rights that parents have over their children. As they note, these rights do not apply by virtue of a genetic connection. Nor does the genetic connection between parents and children make the parent–child relationship exceptionally valuable. My discussion in this paper does not concern parental rights or, specifically, the relationship between people and the children they parent. Rather, it concerns the *characteristics* of the desire for such a relationship, a desire that is often about having genetically related children. In an interesting article that further develops Brighouse and Swift’s account of parental rights, Anca Gheaus argues for the right to parent one’s own biological (i.e., genetically related) children. However, Gheaus’ (2012) article refers to existing rather than desired/future children.

30 One could note that the relationship with a person that is physically or mentally dependent on another for their survival has similar characteristics. However, such a relationship would lack the other characteristics that render the parent–child intimacy special. In addition, unlike dependency due to long-term disability, dependency due to immaturity in children is usually a temporary rather than an existential state.

31 For empirical work on this, see for instance, McMahon (1995), Smith (1999), Habib (2012), Laney et al., (2015).

32 Which, in turn, are mediated by socio-economic status, ethnicity, culture- and religion-specific norms.

33 For instance, some objections could be moved against Dworkin’s theory of equality of resources or its application to the case of state-funded fertility treatment. Burley (1998) considers some of these objections. Others could be moved against the idea that the desire for genetically related children is not akin to other valuable life-projects that one might need state assistance to pursue, as for instance Emily McTernan (2015) argues in her article against the use of public resources to fund fertility treatment.

34 For a discussion of this claim, see Lotz (2016).

35 Elsewhere, I defend this view in the context of fertility treatment during the COVID-19 pandemic. See Cavaliere (2020).
